# Human endogenous retrovirus (HERV) expression is not induced by treatment with the histone deacetylase (HDAC) inhibitors in cellular models of HIV-1 latency

**DOI:** 10.1186/s12977-016-0242-4

**Published:** 2016-02-06

**Authors:** Tara Hurst, Matthew Pace, Aris Katzourakis, Rodney Phillips, Paul Klenerman, John Frater, Gkikas Magiorkinis

**Affiliations:** Department of Zoology, University of Oxford, South Parks Road, Oxford, UK; Nuffield Department of Clinical Medicine, Peter Medawar Building for Pathogen Research, John Radcliffe Hospital, Oxford, UK; Institute for Emerging Infections, The Oxford Martin School, Oxford, UK; Oxford National Institute of Health Research Biomedical Research Centre, University of Oxford, Oxford, UK; Faculty of Medicine, University of New South Wales, Sydney, Australia

**Keywords:** HDAC inhibitors, HIV-1 latency, HIV-1 cure, Endogenous retroviruses, HERV-K (HML-2)

## Abstract

**Background:**

While antiretroviral therapies have improved life expectancy and reduced viral loads in HIV-1-positive individuals, the cessation of treatment results in a rebound of viral replication. This suggests that a reservoir of latently-infected cells remains within these patients, the identity of which is ill-defined and therefore difficult to target therapeutically. Current strategies are aimed at using drugs such as histone deacetylase (HDAC) inhibitors to induce the expression of latent HIV-1 proviruses in order to activate and ultimately eradicate this reservoir of infected cells. One concern with the use of HDAC inhibitors is that they could up-regulate human endogenous retroviruses (HERVs), as well as HIV-1, with potentially pathophysiological consequences.

**Results:**

In this study, we analysed the transcription of HERV genes in HIV-1 latency T cell (J-LAT 8.4) and monocyte (U1) models following treatment with the HDAC inhibitors, vorinostat, panobinostat and romidepsin. We examined the expression of HERV-K (HML-2) *env* and *pol,* as well as the co-opted genes HERV-W *env* (syncytin-1), HERV-FRD *env* (syncytin-2), in these cell lines. Finally, we investigated HERV expression in primary human T cells.

**Conclusions:**

We show that HDAC inhibitors did not substantially increase the transcription of the analysed HERV *env* or *pol* genes, suggesting that histone acetylation is not crucial for controlling HERV expression in these experimental models and in ex vivo primary human T cells. Importantly, this indicates that unwanted HERV expression does not appear to be a barrier to the use of HDAC inhibitors in HIV-1 cure strategies.

**Electronic supplementary material:**

The online version of this article (doi:10.1186/s12977-016-0242-4) contains supplementary material, which is available to authorized users.

## Background

The treatment of HIV-1-infected individuals with antiretroviral therapy (ART) has reduced HIV-1 associated morbidity and improved life expectancy [29]. Despite this, a persisting reservoir of latently-infected cells is the source of rebound in plasma viremia following cessation of ART [8]. An important goal in strategies to cure HIV-1 infection is the depletion of this viral reservoir [8]. One cure approach—‘shock and kill’ [10]—involves first reactivating viral replication and then targeting the infected cells for destruction.

Reactivation of latent HIV-1 has been demonstrated with histone deacetylase (HDAC) inhibitors in clinical trials [4, 5, 13, 28]. The removal of acetyl groups from histones by HDACs leads to transcriptional repression; inhibition of these enzymes is therefore thought to favour gene expression [34]. Vorinostat and panobinostat are hydroxamic acids that bind to zinc, preventing its use by the zinc-dependent HDACs [29], while romidepsin is activated in vivo to produce a zinc-binding thiol [35]. In addition, the use of protein kinase C (PKC) activators, such as phorbol 12-myristate 13-acetate (PMA), has been found to induce HIV-1 expression, via nuclear factor κB (NF-κB) and specificity protein (Sp) transcription factor activation [1].

Given the effect of these agents on HIV-1 provirus expression, we considered whether they could also promote the expression of human endogenous retroviruses (HERVs). Expression is most likely to be relevant and important for intact HERV genes, which would then result in potential health risks as a result of protein activity or an active retroviral replication cycle [24]. HERV-K (HML-2) (HK2) is the most recently active family [26] with intact open reading frames and transcription enhancers within intact long terminal repeats (LTRs). Moreover, two HERV *env* genes (HERV-W *env* and HERV-FRD *env*) have been co-opted by the host and encode proteins (syncytin-1 and syncytin-2, respectively) that are critical for placentation and foetal immunotolerance [12]. We thus designed quantitative RT-PCR assays using molecular beacon probes to HK2 *env* and *pol* genes, as well as the co-opted HERV-W and HERV-FRD *env* genes (Additional file 1: Table S1).

Crucially, HK2 has been shown to be up-regulated by HIV-1 infection [6, 15] as a result of Tat activation of the NF-κB and nuclear factor of activated T cells (NF-AT) transcription factors, which then act on the HERV LTR promoters [16]. Thus, we hypothesise that the reactivation of HIV-1 by HDAC inhibitor treatment could have an indirect effect on HERV expression due to Tat. This indirect effect could be in addition to the potential direct effect of the HDAC inhibitors on HERV expression. The combined direct and indirect effect of HDAC inhibitors could, in theory, lead to an overdrive of HERV activity with potential health risks (e.g. due to genomic instability [7, 24]. We therefore measured the combined direct and indirect effect of HDAC inhibitors on HERV expression in J-LAT8.4 and U1 cells, which are models of HIV-1 latency in cell types that are subject to HIV-1 infection in vivo.

Here, we measured the expression of four HERV targets in cell lines, as well as in primary human T cells. We did not detect substantial up-regulation of HERV transcription, suggesting that the HDACs are not critically involved in controlling HERV expression.

## Results and discussion

### HDAC inhibitors increase H4 acetylation

**Fig. 1:**
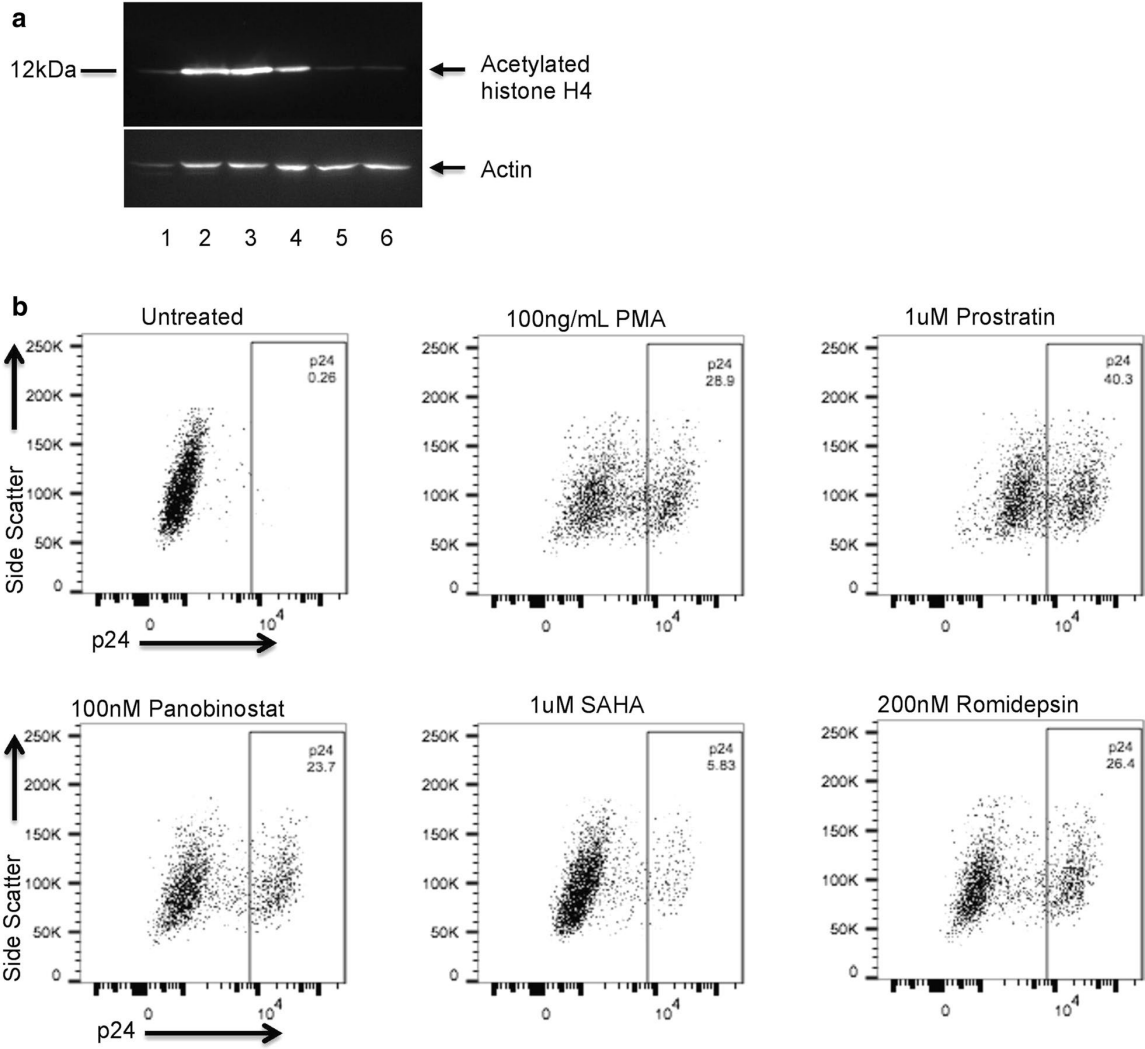
The HDAC inhibitors promote histone acetylation and HIV-1 p24 expression. **a** Treatment of U1 cells with HDAC inhibitors results in increased acetylation of histone H4. The blot was probed with antibody to acetylated H4, followed by HRP-conjugated secondary antibody and enhanced chemiluminescence (ECL) detection. Subsequently, the same blots were reprobed with anti-β-actin antibody. The lanes are (*1*) untreated, (*2*) vorinostat, (*3*) panobinostat, (*4*) romidepsin, (*5*) prostratin and (*6*) PMA. **b** HIV-1 p24 expression is induced by vorinostat, panobinostat, romidepsin, prostratin and PMA, as measured by flow cytometry

To determine if the HDAC inhibitors were functional, total cellular extracts of U1s treated with the inhibitors were analysed by western blotting for acetylated histone H4. The cells were either left untreated or treated with vorinostat (1 μM), panobinostat (0.1 μM), romidepsin (0.2 μM), prostratin (1 μM) or PMA (100 ng/mL) for 24 h. Treatment with the HDAC inhibitors showed an increase in histone H4 acetylation compared to the untreated control (Fig. 1a). The protein kinase C (PKC) activators, PMA and prostratin, did not induce histone acetylation as expected. Importantly, PMA was also tested on the human monocytic cell line, THP1s, and found to induce differentiation into macrophages, resulting in altered morphology (more granular cytoplasm and larger nuclei) and in the cells becoming adherent (data not shown). In addition, HDAC inhibitors induced HIV-1 p24 expression in U1s, as measured by FACS (Fig. 1b). Thus, the HDAC inhibitors were biologically active in the cell types used.

### The HDAC inhibitors vorinostat and panobinostat do not increase HERV expression in J-LAT8.4 or U1 cells

**Fig. 2:**
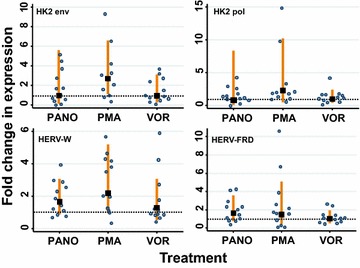
The HDAC inhibitors panobinostat and vorinostat do not increase HERV expression in J-LAT-8.4 cells. The HERVs analysed were: HK2 *env*, HK2 *pol*, HERV-W *env* (syncytin-1) and HERV-FRD *env* (syncytin-2). The fold change in HERV expression following drug treatment was compared to the untreated control (*lines* show 95 % CI) and was calculated relative to GAPDH expression. The doses of the drugs used were vorinostat (1 μM/well), panobinostat (0.1 μM/well), PMA (0.1 μg/μL). The *data points* (*empty circles*) represent the relative fold change in expression normalised with GAPDH (*black squares* show the median and *orange lines* show 95 % CI) for up to three replicates in four independent experiments. A significant change of expression (i.e. higher than the untreated cells) would show the 95 % CI to be higher than and not overlap the *dashed horizontal line* which indicates 1× relative fold change (two-sided test)

We first assessed the relative expression of HERVs in the T-cell latency model, J-Lat 8.4, using the hydroxamic acids, vorinostat and panobinostat, as well as PMA (Fig. 2). The cells were treated with 1 μM vorinostat, 0.1 μM panobinostat or 100 ng/mL PMA for 24 h, then harvested and the RNA extracted. Following rigorous gDNA removal, the RNA was subject to two-step RT-qPCR.

The expression of HK2 *env* and *pol* as well as the syncytins in untreated J-Lat 8.4 cells was much lower than the expression of the housekeeping gene (GAPDH) and had a range of at least 1-log. More specifically, the expression of HK2 *env,* HK2 *pol*, HERV FRD and HERV-W was 2–3 logs lower, 2–5 logs lower, 3–4 logs lower and 3–5 logs lower than GAPDH, respectively. Thus, if HDAC inhibitors were to substantially up-regulate (“resurrect”) HERVs, we would expect to see a substantial change of their expression reducing the gap between their expression and GAPDH by at least 1-log (i.e. at least more than the naturally occurring range).

The expression of HK2 *env* and *pol* were not significantly increased by either vorinostat or panobinostat, with the mean in each case being equal to that of the untreated samples (represented by the dotted horizontal line on the graphs). Panobinostat could increase HK2 *env* expression up to a maximum of fivefold but the majority of samples had values <2-fold. HK2 *pol* expression was increased by a maximum of fourfold, again with the majority of the samples <2-fold. For both HK2 *env* and HK2 *pol*, vorinostat did not induce expression above untreated in most samples; a few samples showed expression between 1- and 4-fold compared to untreated. This variability of the fold expression likely reflects the low copy numbers of HERV RNA. In contrast, PMA induced HK2 *env* expression up to ninefold and HK2 *pol* expression up to 15-fold. By comparison, vorinostat increased HIV-1 expression in CD4+ T-cells by 4.8-fold [2].

Similarly, the expression of the co-opted genes, HERV-W *env* and HERV-FRD *env*, was not substantially increased by panobinostat or vorinostat. The mean values are between 1 and 1.7-fold, with most of the individual samples <2-fold. PMA variably induced the expression of both HERV-W *env* and HERV-FRD *env* up to a maximum of 6- and 10-fold, respectively, compared to the untreated samples (Fig. 2).

**Fig. 3:**
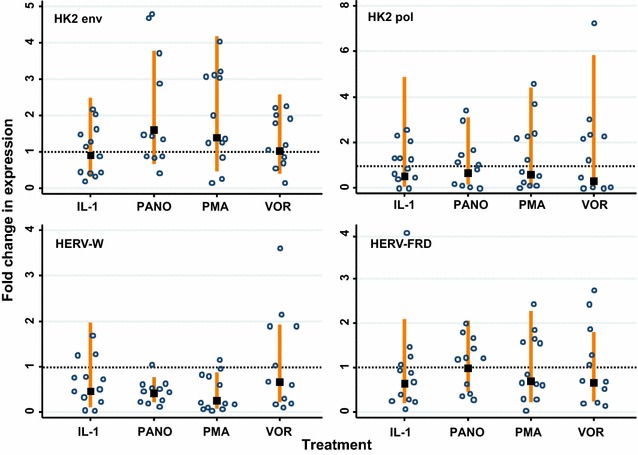
The HDAC inhibitors panobinostat and vorinostat do not increase HERV expression in U1 cells. The HERVs analysed were: HK2 *env*, HK2 *pol*, HERV-W *env* (syncytin-1) and HERV-FRD *env* (syncytin-2). The fold change in HERV expression following drug treatment was compared to the untreated control (*lines* show 95 % CI) and was calculated relative to GAPDH expression. The doses of the drugs used were vorinostat (1 μM/well), panobinostat (0.1 μM/well), PMA (0.1 μg/μL) and IL-1β (10 pg/mL). The *data points* represent the relative fold change in expression normalised with GAPDH (*lines* show 95 % CI) for up to three replicates (*lines* show 95 % CI) in four independent experiments. A significant change of expression (i.e. higher than the untreated cells) would show the 95 % CI to be higher than and not overlap the *dashed horizontal line* which indicates 1× relative fold change (two-sided test)

Next, we treated the monocytic latency model, U1s, with vorinostat, panobinostat and PMA. While panobinostat variably induced HK2 *env* expression, the mean was 1.5-fold (Fig. 3, top panel). Vorinostat did not substantially induce HK2 *env* expression and the mean for the samples was the same as untreated (Fig. 3). HK2 *pol* expression was increased up to threefold with panobinostat treatment in one experiment but was typically increased by 1.5-fold or less. Likewise, vorinostat induced HK2 *pol* expression up to threefold, with a mean of <1; there was one sample that gave up to sevenfold induction but this is likely an outlier given the much lower expression level in the other samples (Fig. 3, top right panel). On the other hand, PMA treatment induced the expression of HK2 *env* by up to 4-fold and HK2 *pol* up to 4.5-fold. The expression of HERV-W *env* and HERV-FRD *env* were not induced by vorinostat, panobinostat or PMA (Fig. 3, bottom panels).

We also treated the U1s with IL-1β since they are monocyte-derived and should respond via the activation of IL-1 receptor (IL-1R)-mediated NF-κB induction. We did so in order to examine if NF-κB activation could induce HERV expression, based on reports that HERVs could have κB response elements in their LTRs [25] and that murine ERVs are responsive to NF-κB following BCR activation [36]. However, IL-1β (10 pg/mL) treatment only induced expression of HK2 *env* and *pol* (Fig. 3, top panels) up to twofold compared to the untreated samples (statistically non-significant). IL-1β did not increase the expression of either HERV-W *env* or HERV-FRD *env* (Fig. 3, bottom panels); in fact, the overall trend suggests that IL-1β downregulates HERV-W and HERV-FRD *env* expression (though statistically non-significant).

### Higher concentrations of the HDAC inhibitors do not up-regulate HERV expression

We next considered whether the concentration of the HDAC inhibitors that we used were too low to induce HERV expression. In the experiments described so far, we have used vorinostat at 1 μM and panobinostat at 0.1 μM, both within the range of concentrations shown to induce HIV-1 expression in cell lines or primary cells [28, 33]. We scaled up the concentrations to examine whether a much higher dose would induce HERV expression. We treated the U1 cells with 1 mM vorinostat or 0.05 mM panobinostat, which represent 1000- or 500-fold higher concentrations compared to those in Figs. 2 and 3, respectively. We noticed a marked level of cell toxicity at these higher doses, with up to a twofold reduction in viable cells after 5 h incubation with either drug compared to untreated cells (data not shown); for this reason, we analysed the RNA from the cells after 5 h rather than 24 h. The short time period of this incubation may limit the detection of changes in HERV expression. However, it has been found that nuc1 nucleosome remodelling following vorinostat treatment for 2 h could occur in as little as 3 h post-treatment [13]. Along with acetylation, nuc1 remodelling is an essential precursor to HIV-1 expression. Further, studies in human primary cells showed vorinostat-induced HIV-1 expression following a 3–6 h of treatment [4].

**Fig. 4:**
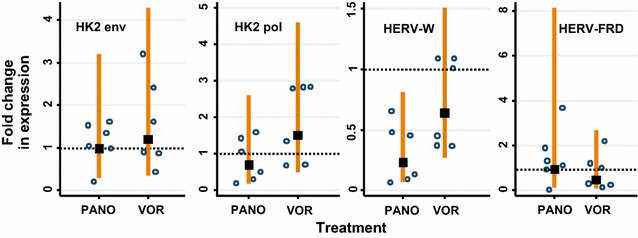
The HDAC inhibitors panobinostat and vorinostat do not increase HERV expression in U1 cells following treatment with higher doses of the drugs for 5 h. The doses of the drugs used in this experiments were: vorinostat 1 μM/well, panobinostat 0.1 μM/well and PMA 0.1 μg/μL. The HERVs analysed were: HK2 *env*, HK2 *pol*, HERV-W *env* (syncytin-1) and HERV-FRD *env* (syncytin-2). The fold change in HERV expression following drug treatment was compared to the untreated control (*lines* show 95 % CI) and was calculated relative to GAPDH expression. The *data points* represent the relative fold change in expression normalised with GAPDH (*lines* show 95 % CI) for up to three replicates in two independent experiments. A significant change of expression (i.e. higher than the untreated cells) would show the 95 % CI to be higher than and not overlap the *dashed horizontal line* which indicates 1× relative fold change (two-sided test)

Despite the higher doses, relative HK2 *env* expression did not substantially increase with either vorinostat or panobinostat (Fig. 4, top left). HK2 *pol* expression increased by up to threefold (statistically non-significant) with vorinostat but not with panobinostat (Fig. 4, top right). The expression of the co-opted genes, were not increased by treatment with vorinostat or panobinostat; strangely, HERV-W *env* appears to be down-regulated by panobinostat (Fig. 4, bottom panels). Thus, the higher doses of the HDAC inhibitors did not remarkably induce HERV expression in the U1s.

**Fig. 5:**
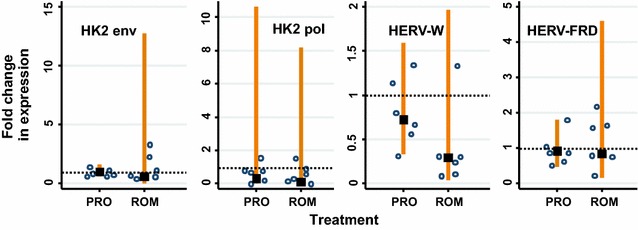
Romidepsin and Prostratin do not increase HERV expression in U1s. The HERVs analysed were: HK2 *env*, HK2 *pol*, HERV-W *env* (syncytin-1) and HERV-FRD *env* (syncytin-2). The fold change in HERV expression following drug treatment was compared to the untreated control (*lines* show 95 % CI) and was calculated relative to GAPDH expression. Romidepsin and prostratin were used at final concentrations of 0.2 and 1 μM, respectively. The *data points* represent the relative fold change in expression normalised with GAPDH (*lines* show 95 % CI) in two independent experiments. A significant change of expression (i.e. higher than the untreated cells) would show the 95 % CI to be higher than and not overlap the dashed horizontal line which indicates 1× relative fold change (two-sided test)

We also considered other classes of HDAC inhibitors, such as the depsipeptide romidepsin. Further, we examined the effect of a different PKC activator, prostratin. Both romidepsin (200 nM) and prostratin (1 μM) were examined for their effect on HERV expression in U1s. In all experiments, the expression of none of the HERVs was increased by either romidepsin or prostratin (Fig. 5).

Overall, the treatment of U1 or J-LAT 8.4 cells with the HDAC inhibitors did not result in significant increases in HERV expression. Doses of the drugs within the range shown to activate HIV-1 expression in latently-infected cell lines [28, 33] were used to treat the cells for up to 24 h but they did not substantially increase HERV expression. Treatment of the cells with significantly higher doses of the drugs did not increase the expression of the HERVs after 5 h.

We next analysed expression of HERVs in uninfected or HIV-1-infected primary CD4^+^ T cells from three individual donors with or without panobinostat treatment. These cells were completely negative for HERV-W *env* and HERV-FRD *env* expression. HK2 *env* was negative for all conditions in all donors, with the exception of HIV-1 infected cells treated with panobinostat in donor 1 (Table 1). Likewise in donor 1, HK2 *pol* was negative in all but the HIV-1-infected cells treated with panobinostat. In contrast, donors 2 and 3 showed a basal level of HK2 *pol* expression in uninfected cells, which was reduced following panobinostat treatment. Likewise, HK2 *pol* was expressed following HIV-1 infection in donors 2 and 3 and this was reduced by panobinostat treatment. For donor 3, the HIV-1-infected cells showed similar levels of HK2 *pol* expression to the uninfected cells; thus, in this individual, HIV-1 expression alone did not induce HK2 *pol*. In combination, HIV-1 infection and panobinostat treatment resulted in a suppression of HK2 *pol* expression, rather than a synergistic effect as we had hypothesised. Overall, the results in primary cells shows undetectable or low levels of the HERVs measured, with the exception of HK2 *pol* expression in one donor. There is donor-to-donor variability, which could be clinically important; some patients could exhibit enhanced HERV expression in response to reactivation of latent HIV-1 and/or HDAC inhibitor treatment and screening for this could be considered. Thus, the results observed in the cell lines are supported by the findings in primary cells since treatment with panobinostat did not enhance HERV expression; indeed, it appears to suppress HK2 *pol* expression.

**Table 1:** HERV expression in primary human T cells

Target	Treatment	Donor 1	Donor 2	Donor 3
HK2 *pol*	Uninfected	0	7.8	3952
	Uninfected + panobinostat	0	5.4	0
	HIV-1	0	186	3783
	HIV-1 + panobinostat	21	21	309
HK2 *env*	Uninfected	0	0	0
	Uninfected + panobinostat	0	0	0
	HIV-1	0	0	0
	HIV-1 + panobinostat	117	0	0

Values given are copies of the HERV target per 1000 copies of GAPDH

It has been reported that HDAC inhibition affects gene expression in 2–20 % of genes [29]. Thus, other mechanisms than histone acetylation/deacetylation regulate HERV expression. The class I HDACs 1, 2 and 3 have been shown to localise to HIV-1 proviruses using chromatin immunoprecipitation (ChIP) [22], which explains why HDAC inhibition can alter HIV-1 expression. Other studies have shown that a number of transcription factors recruit HDACs to the HIV-1 LTR and thereby contribute to latency [9, 18]. In contrast, few studies have looked at the interaction of HDACs with HERV LTRs. One study used ChIP to show that histone H3 acetylation was critical to the expression of HERV-W and HERV-FRD *env* in placental tissues, where they are known to have biological roles [30]. However, in non-placental tissues transcription is suppressed by histone H3 methylation and inefficient splicing [30]. Histone H3 acetylation was detectable in a placental cell line but not in HeLa cells, corresponding to transcriptional activation in the placenta but suppression elsewhere. The study by Trejbalová and colleagues highlights the fact that there could be tissue-specific mechanisms of control of HERV expression. Further to this, and because increased HERV expression has been described in several types of cancer [11], it would be of interest to examine HERV expression following HDAC inhibitor treatment of a panel of uninfected human cell types. Control of HERV transcription by other mechanisms than acetylation has been described, including CpG methylation of HK2 [23]. It is thus possible that the inhibition of HDACs by the hydroxamic acids (vorinostat and panobinostat) or romidepsin has no remarkable effect on HERV expression because either these HDACs are not biologically relevant to the control of HERV transcription or there are additional mechanisms on top of HDACs to control HERV transcription.

One aspect of the study we would like thus to explore further is the effect of combination treatments. Prostratin, a PKC activator, activates HIV-1 in a synergistic manner when used in conjunction with a HDAC inhibitor [29]. Prostratin itself causes nuclear accumulation of the NF-κB p65-p50 heterodimer, the activity of which is inhibited by deacetylation of the p65 subunit by HDAC3 [29]. Inhibition of HDAC3 activity thus permits the acetylation and hyperactivation of NF-κB and the reactivation of HIV-1 from latency [29].

## Conclusions

A key objective of this study was to evaluate HERV expression following treatment with the HDAC inhibitors. Given the use of these drugs in the treatment of certain cancers and the reactivation of latent HIV-1, it is important to consider whether the HERVs are also reactivated. We have previously shown that ERV activity has been suppressed by body size throughout evolution, probably due to higher cancer burden [21], and so increased HERV expression could be detrimental. For example, increased HERV-K expression was detected in germ cell and trophoblastic tumours [17], melanoma cells [27], as well as in the blood of breast cancer [32] and prostate cancer patients [31]. However, whether the expression of HERVs drive tumorigenesis or are the result of the aberrant gene expression in cancers is still undetermined [11]. Further, HERV proteins are not normally detected in healthy human tissues but were detectable following HIV-1 infection in primary human donor cells [19]. Importantly, we do not observe remarkably increased expression of the HERVs with the HDAC inhibitors vorinostat, panobinostat or romidepsin in the context of HIV-1 infection nor in uninfected cells. Other safety concerns, such as the toxicity and mutagenicity of vorinostat [29] remain to be addressed. Finally, our studies have been conducted in cell lines, which have limitations due to being immortalised, clonal cell populations [3]. While these cells might not recapitulate the biology of HIV-1 latency, we have found similar results in primary T cells, suggesting that HDAC inhibitor treatment of HIV-1-infected cells does not lead to a synergistic increase in HERV expression. It will be important to study HERV expression in the context of clinical trials such as RIVER (Research in Viral Eradication of HIV Reservoirs), which employs a ‘kick and kill’ strategy with vorinostat treatment and two different anti-HIV-1 vaccines.

## Methods

### HDAC inhibitors, protein kinase C activators and cytokines

Vorinostat was purchased from Sigma-Aldrich (Poole, UK) and panobinostat was ordered from Cambridge Bioscience (Cambridge, UK). They were each reconstituted in sterile DMSO (Sigma-Aldrich) to a concentration of 20 mM. Romidepsin was purchased from Abcam (Cambridge, UK) and reconstituted at a concentration of 2 mM in DMSO. Prostratin was obtained from Cambridge Bioscience.

Phorbol 12-myristate 13-acetate (PMA) was purchased from Abcam and reconstituted with DMSO at a concentration of 1 mg/mL. Recombinant interleukin-1β (IL-1β) was purchased from Life Technologies (Paisley, UK) and reconstituted at a concentration of 1 μg/mL in pure dH_2_O.

### Cell culture and drug treatments

The following reagents were obtained through the AIDS Reagent Program, Division of AIDS, NIAID, NIH: J-Lat 8.4 cells from Dr. Eric Verdin and U1 cells from Dr. Thomas Folks. More specifically, the J-LAT8.4 cell line is a clone of Jurkat T cells containing one full-length HIV-1 provirus with the green fluorescent protein (GFP) sequence in place of *nef* and a frameshift mutation in the *env* gene [20]. The expression of HIV-1 is low to undetectable in this cell line in the absence of tumour necrosis factor alpha (TNFα) [20]. U1 cells were derived from the promonocytic cell line U937 which were chronically infected with HIV-1 and show limited constitutive activation of HIV-1; recombinant granulocyte/macrophage colony-stimulating factor (GM-CSF) was able to induce expression of HIV-1 in these cells [14]. U1s were examined using restriction enzymes and found to contain two copies of HIV-1 proviral DNA [14].

The cells were cultured in RPMI supplemented with 10 % FBS, 1 % penicillin–streptomycin and 2 mM l-glutamine, all of which were purchased from Sigma-Aldrich. The cells were passaged twice per week at a ratio of 1:5. To test the HDAC inhibitors, the cells were seeded at a density of 5 × 10^5^ cells/well in a 96 well plate.

Unstimulated human primary CD4^+^ T cells were isolated from PBMCs from three separate donors. The cells were either left uninfected or infected with the ×4 tropic LAI strain. For infection, cells were spinoculated for 2 h at 1200 g with viral supernatant, after which the cells were washed twice with supplemented RPMI media and cultured for 48 h in the presence of 1.25 μM saquinavir to prevent spreading infection. After culturing both uninfected and infected cells for 48 h, the cells were either left untreated or treated with 20 nM panobinostat for a further 24 h.

### RNA extraction

The cells were lysed using QIAgen RNeasy kit Buffer RLT and then homogenized using a QIAshredder column. The RNeasy RNA extraction procedure was then followed according to the manufacturer’s protocol (Qiagen, Manchester, UK).

### Removal of genomic DNA

Since there are many copies of HERVs in the genome (for example around 100 almost full-length copies of HK2), it was essential to remove all genomic DNA (gDNA) from the samples. To do so, we used the Turbo DNA-free kit from Life Technologies. Briefly, we treated 10μL of RNA (equal to 1/4.5 of the total extract) with 1 μL DNase and 1.2 μL of the DNase buffer. Next, we incubated the samples at 37 °C for 30 min. We then added 2 μL of the inactivating agent and incubated the samples at 70 °C for 10 min to completely inactivate the DNase (with the inactivating reagent and heat-treatment), as well as protect the RNA from divalent cations (with the inactivating reagent). We pelleted the inactivation agent by centrifugation and the supernatant was reserved as the pure RNA fraction.

We first validated that gDNA was totally removed by negative SYBR green qPCR as well as by negative agarose gel analysis (using HK2 *pol* primers). We also evaluated that the removal of inactivating reagent and that the inactivation of DNase was complete by spiking the samples with 10^5^ copies of our standards and then doing RT-qPCR using a Roche Lightcycler Nano. Quantitative cycle (C_q_) values were as high as expected (data not shown) suggesting that no PCR inhibition occurred due to remnants of the inactivation reagent, but also that DNase activity was no longer present. To evaluate RNA degradation due to the DNase treatments, we also performed two sequential DNase treatments, followed by the inactivation step, and then re-evaluated the RNA in RT-qPCR. C_q_ values after one and two treatments were comparable suggesting that RNA was not degraded throughout the process.

Finally, we diluted the extracted total RNA in either pure or DNase-treated water (1:1 dilution), followed by RT-qPCR and comparing the C_q_ values. We did this to test if removal of divalent cations in the DNase inactivation process was incomplete which would then degrade RNA at high temperature or inhibit reverse transcription. This test also evaluates if removal of DNase is incomplete which could degrade the newly formed cDNA. C_q_ values in the samples diluted with either DNase-treated water or pure water samples were comparable, suggesting that if RNA or cDNA degradation has occurred, it was not detectable.

### Reverse transcription

We used Superscript III reverse transcriptase (Life Technologies) and random hexamers (Bioline, London UK) to reverse transcribe the gDNA-free RNA in a final volume of 20 µL. In parallel, we performed identical reactions lacking the reverse transcriptase (no RT control). The resulting cDNA or no RT control (2.5 µL) were then used in qPCR with Platinum Taq (Life Technologies) in a custom 50 µL master mix. We purchased additional 50 mM MgCl_2_ from Bioline and the deoxynucleotide triphosphate set was purchased from Sigma-Aldrich. The use of the no RT control provided additional assurance that the gDNA had been removed.

### Quantitative PCR

HERVs have multiple copies in the genome, thus conventional PCR (i.e. without probes) might amplify non-specific targets, such as from older non-functional integrations. In order to measure the recently active clade of HK2 and the co-opted copies of HERV-W and HERV-FRD, we designed primers and Molecular Beacons probes using Beacon Designer software (Premier Bio-Soft) and had the oligos and probes synthesized by Sigma-Aldrich. We designed primers and 6-Fam-labelled Molecular Beacon probes to HK2 (*env*, *pol*), HERV-FRD (*env*) and HERV-W (*env*), as well as GAPDH primers and a HEX-labelled Molecular Beacon probe for use as a housekeeping reference gene (Additional file 1: Table S1). We evaluated that the beacons were properly designed through melting curve analyses. We produced DNA standards by PCR using the specific primer sets and a commercial gDNA stock. The resulting PCR amplicons were gel-extracted, purified and verified by Sanger sequencing, then they were quantified with the Quant-iT™ PicoGreen^®^ dsDNA Assay Kit (Thermo Fisher Scientific), serially diluted and used to construct standard curves from 1 to 10^6^ copies/reaction. We included standards in each qPCR reaction as controls and to evaluate the sensitivity of the reaction.

Further, to ensure that our results were not biased by possible effects of the drugs on GAPDH, we evaluated the cDNA with two other housekeeping genes. The human endogenous control kits for glucoronidase (GUSB) and 18S rRNA were purchased from Life Technologies. The correlations between GAPDH and GUSB or GAPDH and 18S rRNA were very strong (R^2^ = 0.94 or R^2^ = 0.89, respectively), suggesting that the normalisation using GAPDH was relatively unbiased.

### Acetylated histone H4

We seeded J-Lat 8.4 or U1 cells at a density of 1 × 10^6^ cells/well in 6-well plates. The following day, we treated cells with the HDAC inhibitors. The cells were harvested 24 h later by centrifugation and the pellets were washed once with PBS. We then lysed the cells in lysis buffer (137 mM NaCl, 20 mM Tris–HCl, 1 mM EDTA, 0.5 % Triton-X 100) supplemented with protease inhibitor cocktail and N-octylglucoside on ice for 30 min. Subsequently, we centrifuged the lysates for 10 min at 10,000*g* to pellet the nuclei and the supernatant was reserved for analysis. SDS-containing sample buffer was added to the lysate to a final concentration of 1×. Next, we boiled the samples for 5 min, chilled them on ice and centrifuged briefly.

We then analysed the samples by discontinuous SDS-PAGE (12 % resolving gel), followed by transfer for 45 min at 100 V onto 0.22 μM PVDF. The membrane was blocked in Starting Block reagent (Thermo Scientific, Basingstoke, UK), which was also used as the antibody diluent. We used a rabbit polyclonal antibody to acetylated histone H4 from Santa Cruz Biotechnology Inc. (Insight Biotechnology, Wembley, UK), at a concentration of 1/1000 in the diluent. The blots were incubated overnight in primary antibody at room temperature with constant rocking. The next day, we washed the blots three times in PBS with 0.05 % Tween 20 (Fisher Scientific). We then added the secondary anti-rabbit-HRP antibody at a 1/4000 dilution for 1 h at room temperature. The blot was then washed twice with PBS-Tween and once with PBS alone. We then developed the blot using using Novex ECL and visualised using the Syngene G:box system. The blot was subsequently reprobed with a rabbit polyclonal antibody to β-actin (Santa Cruz Biotechnology Inc.) at a concentration of 1/1000 to show equal loading.

### Intracellular p24 staining

For intracellular p24 staining, cells were first stained with the LIVE/DEAD fixable near-IR dead cell stain kit (Life Technologies) as per the manufacturer’s protocol, then fixed with 2 % paraformaldehyde for 30 min. Cells were subsequently washed and then simultaneously permeabilised with 0.05 % saponin and stained using the KC57 PE antibody (Beckman Coulter) for 40 min. HIV-1 p24 levels were then measured by flow cytometry.

### Calculation of the Mean and 95 % Confidence Intervals (CI) of the gene expression

The fold change of expression is a positive real number, with asymmetric long-tailed distribution, which we assume follows a log-normal distribution. We log-transform the fold change and calculate the 95 % CI of the log-transformed mean according to a normal distribution. Each cell-culture experiment had numerous replicates (2 or 3) and was repeated multiple times (up to 5). Thus to calculate the variance of the mean, we do not pool all the replicates from different cell culture experiments, but instead we calculate the variance for each experiment and then combine the variance metrics as follows: suppose we have two experiments containing *n*_1_ and *n*_2_ replicates with means $\overline{X}_{1}$ and $\overline{X}_{2}$, and variances $S_{1}^{2}$ and $S_{2}^{2}$. If $\overline{X}_{c}$ is the combined mean and $S_{c}^{2}$ is the combined variance of *n*_1_ + *n*_2_ replicates, then the combined variance is given by:

$$S_{c}^{2} = \frac{{n_{1} S_{1}^{2} + n_{2} S_{2}^{2} + n_{1} \left( {\overline{X}_{1} - \overline{X}_{c} } \right)^{2} + n_{2} \left( {\overline{X}_{2} - \overline{X}_{c} } \right)^{2} }}{{n_{1} + n_{2} }}$$

and

$$\overline{X}_{c} = \frac{{n_{1} \overline{X}_{1} + n_{2} \overline{X}_{2} }}{{n_{1} + n_{2} }}$$

The confidence interval of the log-transformed values is calculated as $\overline{X}_{c} \pm 1.96\sqrt {\frac{{S_{c}^{2} }}{{n_{e} }}}$ where $\sqrt {\frac{{S_{c}^{2} }}{{n_{e} }}}$ is the standard error of the log-transformed mean and *n*_*e*_ is the number of the individual cell culture experiments.

## Additional file

10.1186/s12977-016-0242-4 Sequences of primers and Molecular Beacon probes used in qPCRs. All HERV probes were labelled with 6-Fam (5’) and BHQ1 (3’), while GAPDH probe was labelled with HEX (5’) and BHQ1 (3’).

## Abbreviations

HDAC: histone deacetylase
HERV: human endogenous retrovirus
LTR: long terminal repeat
