# Supplementary material for: Human endogenous retrovirus (HERV) expression is not induced by treatment with the histone deacetylase (HDAC) inhibitors in cellular models of HIV-1 latency
Source: Retrovirology. 2016 Feb 6;13:10. doi: 10.1186/s12977-016-0242-4 (PMC4744380; doi:10.1186/s12977-016-0242-4)
Supplement: Supplementary file 1 — 10.1186/s12977-016-0242-4 Sequences of primers and Molecular Beacon probes used in qPCRs. All HERV probes were labelled with 6-Fam (5’) and BHQ1 (3’), while GAPDH probe was labelled with HEX (5’) and BHQ1 (3’). [file 12977_2016_242_MOESM1_ESM.docx]

**SUPPLEMENTARY INFORMATION**

**Table S1**. Sequences of primers and Molecular Beacon probes used in qPCRs. All HERV probes were labelled with 6-Fam (5’) and BHQ1 (3’), while GAPDH probe was labelled with HEX (5’) and BHQ1 (3’).

| Target | Oligo | Sequence (5’-3’) |
| --- | --- | --- |
| HK2 *Env* | Sense Primer  Antisense primer  Molecular Beacon | AGTAGATTCACTTATCACATGGT  GTAATATCACCGCACTATTGGCC  CGCGATCCCCAAGGAAATTCCCAAAGAATCAGATCGCG |
| HK2 *Pol* | Sense Primer  Antisense primer  Molecular Beacon | AATTGACTGTTAYACATTTCTRC  CCGAATCCAATTAATATCTCC  CCGAGCCCATCTGATAAGATCCAAACCTCTACTCGGCTCGG |
| HERV-FRD *Env* | Sense Primer  Antisense primer  Molecular Beacon | TCATTTCCATATCTCTACATGCC  GGTACAAGTTCCAGTCCAGTTAC  CGCGCTGTGGCCAGTCGATTCACGCGCG |
| HERV-W *Env* | Sense Primer  Antisense primer  Molecular Beacon | AGCAATACTACATACACAAC  TAGATTCTGAAGAGCCATTC  CGCGATCCCTCCCACACAAATAGTCTGCGATCGCG |
| GAPDH | Sense Primer  Antisense primer  Molecular Beacon | ACAGCCTCAAGATCATCAGCA  ATCACGCCACAGTTTCCCG  CGAGTCCTTCCACGATACCACTCG |
